# Multi‐Omics Reveals Mechanisms of Metabolic Rejuvenation in Aged Mice and Pre‐Frail Older Men by Losartan

**DOI:** 10.1111/acel.70498

**Published:** 2026-04-22

**Authors:** Michael R. Bene, Cissy Zhang, Reyhan Westbrook, Mariann Gabrawy, Ruth Marx, Yuqiong Wu, Mohammed Khadeer, Ceereena Ubaida‐Mohien, Luigi Ferrucci, Ruin Moaddel, Anne Le, Jeremy D. Walston, Jessica L. Lee, Peter M. Abadir

**Affiliations:** ^1^ Division of Geriatric Medicine and Gerontology, Department of Medicine The Johns Hopkins University School of Medicine Baltimore Maryland USA; ^2^ Gigantest Baltimore Maryland USA; ^3^ Biomedical Research Centre, National Institute on Aging National Institutes of Health Baltimore Maryland USA; ^4^ Division of Geriatric and Palliative Medicine, Department of Internal Medicine, McGovern Medical School The University of Texas Health Science Center at Houston Houston Texas USA

## Abstract

Aging is associated with significant alterations in systemic metabolism across species. We employed targeted metabolomics to investigate the effects of losartan, an angiotensin II receptor blocker, on the serum metabolome of aged mice and pre‐frail older men. Losartan treatment resulted in a shift in serum metabolome aging signature to a more youthful state. This rejuvenation effect appears to be contingent on the presence of functional angiotensin II receptors, with receptor knockout mice showing no rejuvenation effect with treatment. Additionally, we observed a similar rejuvenation effect of losartan in the cardiac proteome of aged mice, with the most pronounced changes occurring in proteins involved in oxidative phosphorylation. While our study did not encompass a full lifespan analysis, in alignment with previous reports of lifespan extension in other models, we noted a statistically significant improvement in survival among geriatric mice treated with losartan. In parallel, we analyzed serum metabolomics data from pre‐frail older men from a phase 2 randomized placebo‐controlled trial of losartan, which indicated a dose‐dependent metabolic rejuvenation effect. Correlation network analysis revealed divergent aging effects between species, with mice exhibiting broad decreases in metabolite concentrations and humans showing increases, particularly across lipid species. Principal component analysis further highlighted a global shift in metabolite levels, potentially linked to changes in lipoprotein metabolism, plasma volume, and amino acid metabolism with age. In summary, our results suggest that losartan can partially reverse age‐related metabolomic changes in both male mice and humans, with distinct species‐specific responses.

## Graphical abstract

Losartan partially reversed serum metabolomic aging signatures in both aged mice and pre‐frail older men, and this effect required functional angiotensin II receptors in mice. Cross‐species analyses suggest aging shifts circulating metabolites in opposite directions in mice versus humans, particularly among lipids, with losartan partially normalizing these patterns.

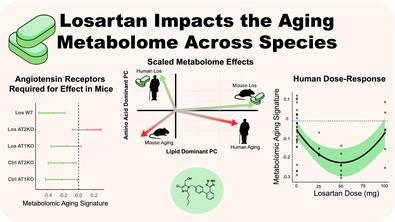

## 1 Introduction

The renin‐angiotensin‐aldosterone system (RAAS) has been investigated for its role in aging for decades. A critical regulator of blood pressure and fluid balance, the importance of the RAAS for cardiovascular health is well studied. Research by Benigni et al. suggested that genetic disruption of the angiotensin II receptor type 1 (AT_1_R) could extend lifespan in mice and later that single‐nucleotide polymorphisms of the same gene were associated with extreme longevity in humans (Benigni et al. 2009; Benigni et al. 2012). Losartan, the first angiotensin receptor blocker (ARB) and a selective, competitive, AT_1_R antagonist, is one of the most prescribed medications, primarily for the treatment of hypertension (Fuentes et al. 2018). Like genetic modification of AT_1_R, losartan treatment has also been associated with aging and longevity. A study by Basso et al. reported that long‐term treatment with losartan significantly extended lifespan in rats (Basso et al. 2007). Further preclinical evidence in mice suggests losartan may protect against disuse atrophy in sarcopenia and improve markers of inflammation and oxidative stress (Burks et al. 2011; Lin et al. 2014). A phase II randomized placebo‐controlled trial of losartan in pre‐frail older adults found that higher concentrations of losartan metabolites correlated with improved physical function and lower odds of frailty (Lee et al. 2022). Given the importance of the RAAS in critical physiological systems, as well as losartan's many reported impacts on aging organisms, we therefore sought to evaluate the impact of losartan treatment on systemic metabolism in both aged male mice and pre‐frail older men.

## 2 Methods

### 2.1 Animals

This study was approved by the Johns Hopkins Animal Care and Use Committee (ACUC). A total of 106 male mice were used for experiments, with 73 mice used for metabolomic and proteomic assessment and 33 in the survival study. Of the 73 mice used for omics analysis, 47 were C57BL/6J wild type (WT) mice (Jackson Laboratories, Bar Harbor, Maine), including 17 young 4‐month‐old mice and 30 24–28‐month‐old mice. The remaining mice used for omics analysis were 13, 24–28 month old AT1R knockout (AT1R−/−) (Jackson Laboratories, Bar Harbor, Maine) mice and 13, 24–28 month old AT2R knockout (AT2R −/−) mice (supplied by our collaborator Dr. Tedashi Inagami, Vanderbilt University, TN) (Tanaka et al. 1999; Ichiki et al. 1995; Ito et al. 1995). Four weeks before sacrificing for omics assesment 27 of the 73 mice received losartan (0.9 g/Liter for aged mice, Cozaar, Merck) ad libitum in their drinking water for 4 weeks: 16 WT old mice, 5 AT_1_KO, and 6 AT_2_KO. For the survival analysis, 33 30‐month‐old WT mice were divided into two groups, with 19 mice receiving 4 weeks of losartan 0.9 g/L ad libitum in drinking water and the other 14 receiving no losartan exposure.

Mice were housed in 75 in^2^, autoclave sterilized, high‐temperature polycarbonate shoebox cages in ventilated racks (Allentown Inc.) containing autoclaved corncob bedding (Harlan Teklad), autoclaved mouse chow 2018SX (Harlan, Teklad), and reverse osmosis–filtered hyperchlorinated water dispensed through an in‐cage automatic watering system (Edstrom Industries). Rooms were maintained at 22°C ± 3.6°C on a 14‐h light/10‐h dark cycle with automated monitoring by Siemens Building Technologies Inc. Cages were changed every 2 weeks in laminar airflow change stations (The Baker Co.) with surface cleaning and disinfection with MB‐10 disinfectant (Quip Laboratories Inc.). All caging was sanitized by automatic cage washing systems and autoclaved before use.

### 2.2 Metabolomics

Metabolites were extracted, and concentrations were assessed using the AbsoluteIDQ kit p180 (Biocrates Life Science AG) following the manufacturer's protocol for the API5500 liquid chromatography‐mass spectrometry (LC–MS) System (AB SCIEX) running with Analyst 1.5.2 software equipped with an electrospray ionization source, a Shimadzu CBM‐20A command module, a LC‐20AB pump, a Shimadzu SIL‐20 AC‐HT autosampler, and a CTO‐10Ac column oven heater at Gigantest (60). Briefly, 10 μL of plasma‐EDTA samples were pipetted onto the center of the spots in each well of a 96‐well Biocrates kit. The samples were dried with a Microvap 118 from Organomation Associate nitrogen evaporator at room temperature (RT) for 30 min. A total of 50 μL of 5% PITC reagent was added and incubated for 20 min, and the plate was dried under nitrogen for 1 h. A total of 300 μL of 5 mM ammonium acetate in methanol was added to each well and incubated at RT on a shaker (450 rpm) for 30 min. The plate was then centrifuged at 100 g for 2 min, resulting in about 350 μL of sample extracts in the capture plate; 50 μL of each sample was transferred to the empty 96–deep well plate; and 10 μL of each sample was transferred from the capture plate to the empty 96–deep well plate labeled as “Use for LC.” The extracts were diluted for LC by adding 450 μL of 40% methanol (in HPLC‐grade water) to each well. The extracts were diluted for flow injection analysis (FIA) by adding 490 μL of FIA running solvent (Biocrates solvent diluted with HPLC grade methanol). The LC–MS plate was run first, with 10 μL injected onto the Eclipse XDB C18, 3.5 μm, and 3.0 × 100 mm with a Phenomenex C18 Security Guard Cartridge, 3.0 mm internal diameters (ID). The mobile phase consisted of solvent A (water containing 0.2% formic acid) and solvent B (acetonitrile containing 0.2% formic acid), with the following gradient: 0–0.5 min 0% B, 5.5 min 95% B; 6.5 min 95% B; 7.0 min 0% B; 9.5 min 0% B. Evaluation of the samples was carried out using the MetIDQ software. The FIA plate was run with 20 μL injection directly into the MS at a flow of 30 μL/min with water/acetonitrile (1:1) containing 0.2% formic acid as the mobile phase, with the following flow rate program: 0–1.6 min 30 μL/min; 2.4 min 200 μL/min; 2.8 min 200 μL/min; and 3.00 min 30 μL/min. Concentrations were calculated using the Analyst/MetIDQ software. PITC, ammonium acetate, water, methanol, and acetonitrile (LC–MS grade) were purchased from MilliporeSigma. QA measurements are relative and represented by the AUC. Of the 186 preconfigured metabolites and ratios in the kit, 122 metabolites were quantifiable in our mouse serum samples and 160 in the human serum samples, with 118 overlapping between species.

### 2.3 Proteomics

Protein was isolated as previously described (Foster et al. 2016). Proteins were identified by searching mouse RefSeq and SwissProt databases using Mascot as implemented in Proteome Discoverer v2.3. Parent ion and MS/MS ion search tolerances were 6 ppm. TMT modification was set as a fixed modification. Cysteine carbamidomethylation and methionine oxidation were included as variable modifications. Analysis of mouse cardiac proteome was performed as previously described (Nidadavolu et al. 2021). Mean abundance differences in proteins, for which *p* < 0.05, were deemed significant. A total of 1220 proteins were identified in whole cell, 412 proteins in plasma membrane, and 607 proteins in the mitochondrial fraction.

### 2.4 Clinical Study

Samples were taken from human subjects involved in a previously described phase II placebo‐controlled trial for exploratory analysis. Only male participants' data were used to match the all‐male mouse analysis and due to the limited number of samples from female participants. All subjects were 70 years old or older and classified as pre‐frail by the physical frailty phenotype criteria at baseline. The clinical trial was registered with ID NCT01989793 on clinicaltrials.gov.

### 2.5 Analysis

All analyses were conducted using R 4.4.1 unless otherwise stated. Metabolomics data were log2 transformed for analysis. A threshold of ±3 in mean metabolite *z*‐score was used to identify outlier samples for both mouse and human metabolomics data. For mouse data, Log2FC and false discovery rate (FDR) corrected *p*‐values were calculated using ‘Group’ and ‘BodyWeight’ as a covariate, and a linear model was fitted using the lmFit function from the limma package. For human data, ‘Treatment’, ‘BMI’, and ‘ID’ were used to account for the longitudinal design. Pairwise contrasts between groups were defined and analyzed with empirical Bayes moderation to assess differences using the eBayes function. Correlations between group effects were calculated using the “cor.test” function. Metabolite class values were calculated by summing the values of all metabolites in each class. For mice, the lmer function from the lme4 package was used to model the effects of age, genotype, losartan treatment, and body weight as fixed effects and individual mouse as a random effect on metabolite class levels (models: metaboliteclass ~1 + Age + BW + Genotype*Treatment+(1|ID)). The paintomics 4 v1.0 webapp was utilized to perform pathway network analysis of the proteomics data using KEGG pathways. Subsequently, cystoscope was used to generate protein–protein‐interaction networks of measured proteins in the oxidative phosphorylation KEGG pathway using the STRING database. To evaluate potential changes to cell composition in the proteomics data, single‐cell marker genes for heart cell types were utilized from the DISCO database for gene set enrichment analysis performed with the fgsea function. Survival analysis was performed using the survfit function and plotted with ggsurvplot from the survminer and survival packages respectively, and the log‐rank test was used to determine significance.

For the human metabolomics aging signature, the β‐coefficient for age from Verri Hernandes et al. data and model was used to establish age‐related changes to metabolites (Verri Hernandes et al. 2022). To determine changes to aging signature at the individual level, each participant's change at a given point was compared to their own baseline measure to account for individual response, and this log2FC was then correlated to the age signature. To test dose–response effects, a linear mixed effects model was used with 1st, 2nd, and 3rd order polynomial models compared and 2nd order selected based on lowest deviance (AgingSignature ~1 + Dose + I (Dose^2) + (1|ID)). Additionally, to assess dose dependent effects on metabolite classes, a similar model was used (metaboliteclass ~1 + Dose + I (Dose^2) + BMI+(1|ID)). To generate the correlation network, the cor.test function was used to generate spearman correlations between all treatments and a correlation network was constructed using the igraph package. Edge properties were determined by correlation strength (thickness, color) and significance (opacity), and plotting was done using a multidimensional scaling layout. Lastly, principal component analysis was performed using prcomp on scaled effects to account for large differences in scales between β‐age used for human aging and log2FC used for mouse aging and losartan effects.

## 3 Results

### 3.1 Losartan Mildly Opposes Serum Metabolome Aging Signature in Mice

**FIGURE 1:**
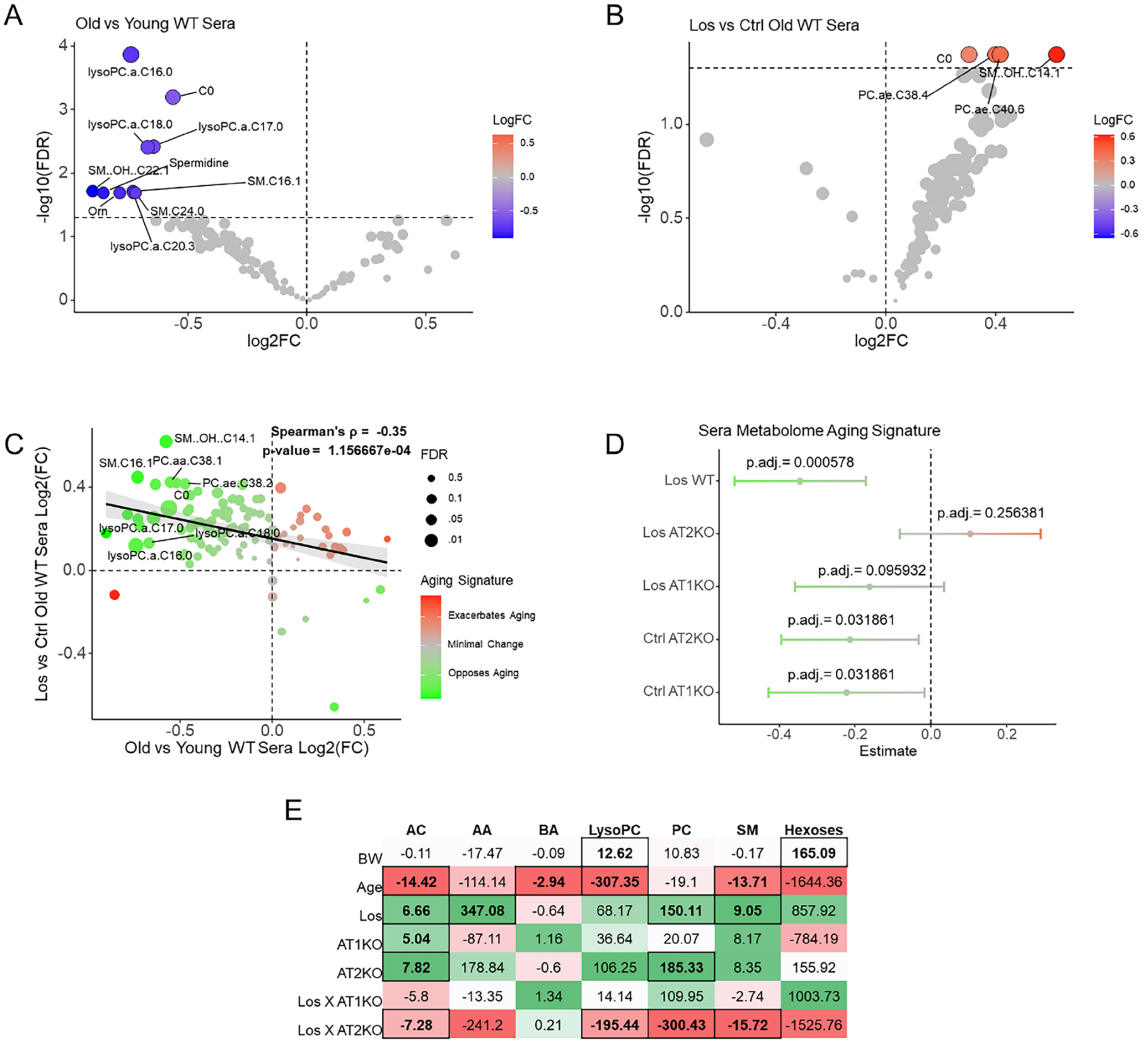
Effects of age and losartan on the serum metabolome of mice. (A) Volcano plot of serum metabolites from old vs. young WT mice (B) serum of los treated vs. control old mice Analyzed using limma‐ebayes with bodyweight as a covariate (C) Bubble plot with x‐axis position being old vs. young WT mouse sera log2FC (aging effect) and y axis being Los vs. control WT Mouse sera Log2FC (losartan effect) (C) Forrest plot of Spearman's rho correlations from comparisons between losartan treated wild type old mice (Los WT), losartan treated AT2KO old mice (Los AT2KO), losartan treated AT1KO old mice (Los AT1KO), AT2KO old mice, and AT1KO old mice with wild type aging metabolome signature, BH‐corrected *p* values (E) Heatmap of the estimated effects from lmer linear mixed‐effects model of bodyweight, age, genotype, losartan treatment, and interactions on overall metabolite level by class. AA, amino acids; AC, acylcarnitine's; BA, biogenic amines; LysoPC, lysophosphatidylcholines; PC, phosphatidylcholines; SM, sphingomyelins. Significance of individual metabolites evaluated with limma ebayes and FDR adjusted *p*‐values. Bubble sizes correspond to FDR corrected Fischer's combined *p*‐values across both comparisons for each metabolite, with those with the lowest significant *p*‐values labeled. Color of sera aging signature indicates concordance (red) or discordance (green) between y‐axis and x‐axis value of metabolite. Young WT *n* = 7, old WT Ctrl *n* = 10, AT1KO Ctrl *n* = 8, AT2KO Ctrl *n* = 7, Los WT *n* = 14, Los AT1KO *n* = 5, Los AT2KO *n* = 6.

To evaluate the metabolic effects of losartan in aging mice, we conducted targeted metabolomics on serum samples from young (4 months) and aged (24–28 months) male C57BL/6J mice (WT). We also included aged mice lacking angiotensin II receptor type 1 (AT_1_KO) and type 2 (AT_2_KO) to separate the angiotensin II receptor‐dependent and independent effects of losartan. Subgroups of aged WT, AT_1_KO, and AT_2_KO mice were treated with losartan (0.9 g/L) in their drinking water for 4 weeks prior to tissue collection. We identified ten differentially abundant metabolites in the serum of aged compared to young WT mice, including decreased levels of carnitine (C0), ornithine, spermidine, and several lipid species including Lysophosphatidylcholines and sphingomyelins (Figure 1A). Notably, no individual metabolites significantly increased in the serum of aged mice. In contrast, losartan treatment in aged WT mice was associated with significantly higher levels of carnitine (C0) and lipids including phosphatidylcholines and sphingomyelins (Figure 1B). We used the aging‐related changes in the serum metabolome as an “aging signature” to compare the global effects of losartan across all metabolites using Spearman's *ρ* correlation (Figure 1C). Specifically, by comparing the FC observed with age in each metabolite to the FC observed with losartan treatment we aimed to provide a summary global assessment of how metabolite shifts from losartan relate to those from normative aging. This analysis revealed a significant negative correlation between the aging metabolomic signature and losartan treatment (*ρ* = −0.35, *p* = 1.15e‐4), indicating that losartan shifted the serum metabolome of aged WT mice in opposition to the metabolic aging signature. We also evaluated the impacts of AT_1_KO and AT_2_KO, with or without losartan treatment, using the same correlation method (Figure 1D). The results showed that both AT_1_KO and AT_2_KO alone produced metabolome shifts that opposed the aging metabolomic signature, while losartan treatment in AT_1_KO mice exhibited a weaker negative correlation with the aging metabolomic signature that did not reach statistical significance (*p*.adj = 0.09). Interestingly, losartan treatment in AT_2_KO mice appeared to negate the metabolome shift observed with either losartan treatment or AT_2_KO alone, suggesting that functional AT_2_R are necessary for losartan's effects. Finally, we assessed the influence of body weight, age, losartan treatment, and genotype on metabolite abundance by class (Figure 1E). Age was associated with a trend toward reduced levels across all metabolite classes, with significant effects noted in acylcarnitines, biogenic amines, lysophosphatidylcholines, and sphingomyelins. Conversely, losartan treatment generally increased the abundance of most metabolite classes, with significant effects observed in acylcarnitines, amino acids, phosphatidylcholines, and sphingomyelins.

### 3.2 Losartan Moderately Opposes Cardiac Proteome Aging Signature Mice

**FIGURE 2:**
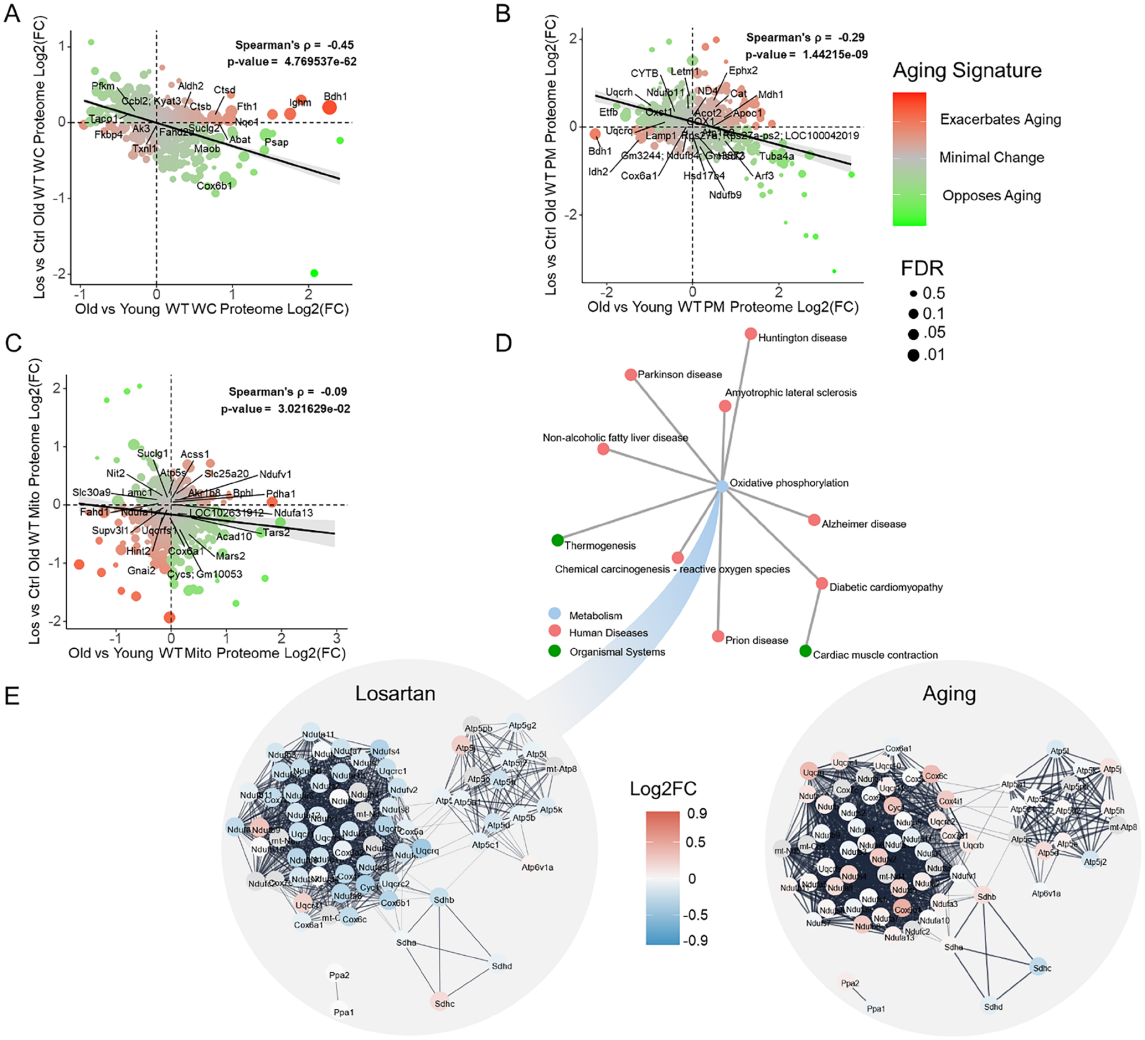
Losartan opposes aging proteome in heart with network pathway analysis identifying OXPHOS as central hub of effect. Bubble plots with x‐axis position being old vs. young WT mouse log2FC (aging effect) and y axis being Los vs. control WT MouseLog2FC (losartan effect) from (A) Whole Cell Heart Tissue (B) Plasma Membrane fraction of heart and (C) isolated mitochondrial fraction from heart. (D) Network plot of significantly altered pathways using whole cell proteome from heart performed using paintomics 4 v1.0 (E) protein–protein interaction network for proteins in the KEGG OXPHOS pathway using STRING with proteins colored by log2FC comparing losartan treated old vs. control old mice (Losartan) and control old to young (Aging). For (A–C) Bubble sizes correspond to FDR corrected Fischer's combined *p*‐values across both comparisons from limma ebayes for each protein, with those with the lowest significant *p*‐values shown. Color of heart aging signature indicates concordance (red) or discordance (green) between y‐axis and x‐axis value of protein. Young WT *n* = 12, old WT Ctrl *n* = 12, Los WT *n* = 12.

To assess whether losartan induces similar alterations in tissue and across other “omics” layers, we conducted targeted proteomics on cardiac tissue from young, aged, and losartan‐treated aged mice. Like the metabolomics data, we used the young and aged cardiac tissue samples to construct a cardiac proteome aging signature. Of the 1220 detected proteins in whole cell cardiac tissue, 247 individual proteins showed significant differences in abundance between young and old samples, demonstrating substantial age‐related remodeling of the cardiac proteome (Table S1). We analyzed the impact of losartan on the aging cardiac proteome by performing a correlation analysis of the cardiac proteome aging signature (X‐axis) and losartan treatment effect (Y‐axis) on whole‐cell cardiac tissue (Figure 2A). This analysis revealed a significant negative correlation, which was stronger than that observed in the serum metabolome, suggesting losartan exerts a more pronounced effect on the cardiac proteome aging signature (*ρ* = −0.45, *p* = 4.77e‐62). To investigate the potential sub‐cellular effects of losartan, we conducted a similar analysis on the plasma membrane and mitochondrial fractions (Figure 2B,C). Both fractions exhibited a significant negative correlation between losartan treatment and fraction‐specific cardiac proteome aging signature; however, the effect was slightly weaker in the plasma membrane and notably weaker in the mitochondrial fraction. We then performed pathway analysis to identify the pathways affected by losartan in the cardiac whole‐cell proteome. The results highlighted oxidative phosphorylation (OXPHOS) as a central hub for losartan's effects (Figure 2D). To further explore the impact of losartan on OXPHOS, we constructed a protein–protein interaction network using STRING based on the proteins included in the OXPHOS pathway defined by KEGG (Figure 2E) (Szklarczyk et al. 2023). The results indicated a general reduction in the abundance of OXPHOS‐related proteins following losartan treatment, contrasting with observed higher OXPHOS protein abundance in the aged control cardiac samples compared to young samples.

### 3.3 Transient Losartan Treatment Improves Survival of Geriatric Mice

**FIGURE 3:**
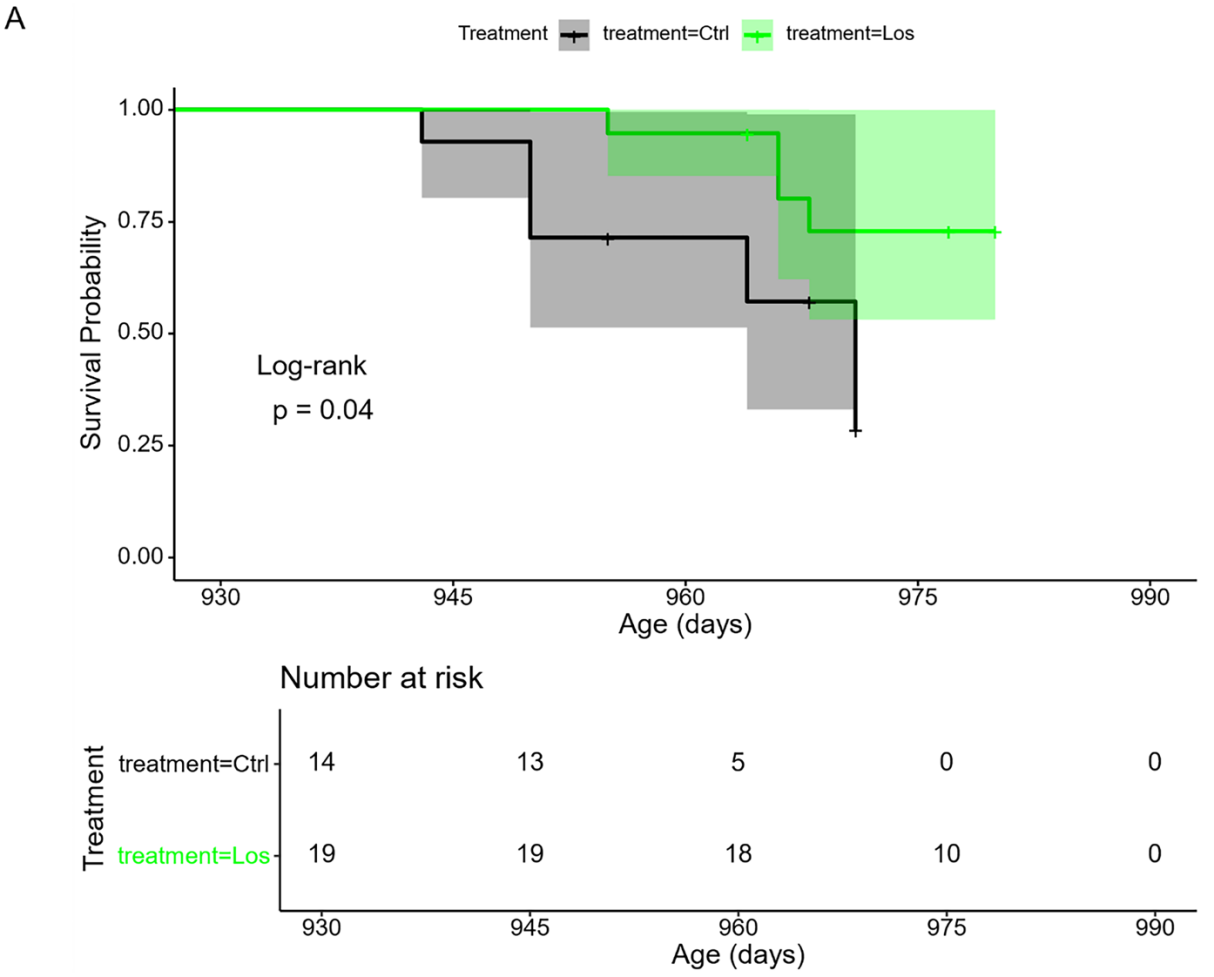
Losartan treatment improves survival of aged mice. (A) survival curve of control and losartan group WT mice, significance by log‐rank test. Total *n* = 33, 19 losartan treated for 4 weeks starting at 30 months and 14 control.

Given the capacity for losartan treatment to impact aging signatures in the metabolome and proteome of aged mice, we evaluated whether treatment of geriatric (30‐month‐old) mice with losartan was associated with improved survival. 33 male WT mice were split into two groups, with 19 receiving 4 weeks of losartan treatment and 14 having no losartan exposure. Though the experiment was not designed as a full lifespan study, we notably observed a significant improvement in survival by log‐rank test, even within the short, 2‐month observation period before sacrifice of the animals (Figure 3A).

### 3.4 Losartan Dose‐Dependently Opposes Serum Metabolomic Aging Signature in Pre‐Frail Older Men

**FIGURE 4:**
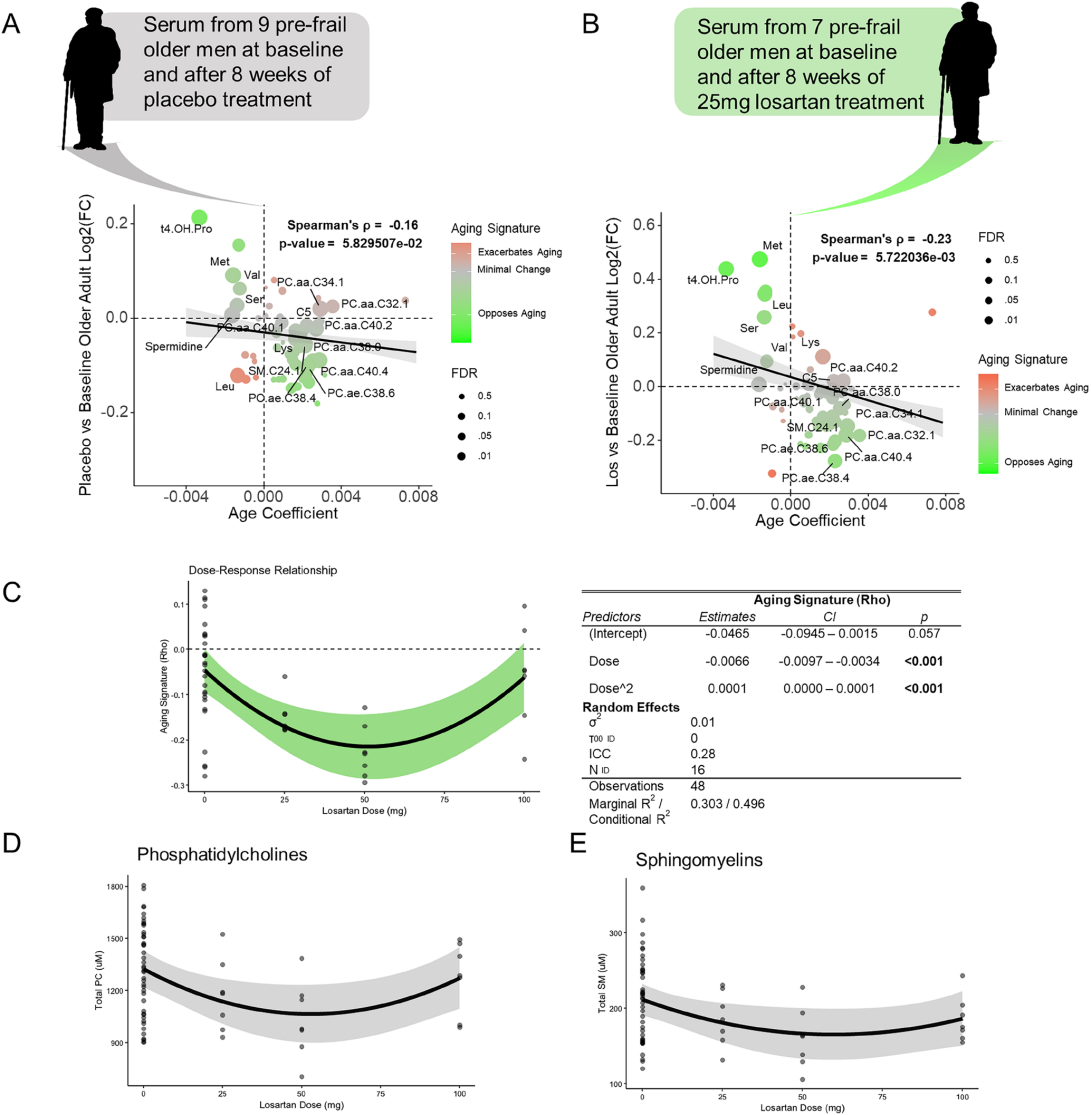
Losartan treatment weakly opposes serum metabolome aging in pre‐frail older men. Bubble plots with x‐axis position being coefficient of age from Verri Hernandes et al. (aging effect) and y axis being post‐treatment vs. baseline (treatment effect) from (A) placebo group (B) losartan group (C) Individual correlations between dose vs. baseline and age effect (aging signature‐rho) by dose, modeled with a linear‐mixed effects model with dose treated as second order polynomial fixed effects and subject ID as random effects (D) Dose response of total (summed) phosphatidylcholines modeled with a linear‐mixed effects model with dose treated as second order polynomial and BMI as fixed effects and subject ID as random effects (E) Dose response of total (summed) sphingomyelins modeled with a linear‐mixed effects model with dose treated as second order polynomial and BMI as fixed effects and subject ID as random effects. Placebo *n* = 9, losartan treatment *n* = 7.

While the effects of losartan in aged mice suggest potential gerotherapeutic benefits, it remains uncertain whether these impacts would translate to humans. To investigate this, we utilized targeted metabolomics data from the previously described phase 2 randomized controlled trial of losartan in pre‐frail older adults, employing the same platform used in our mouse experiments. To facilitate a global “aging signature” analysis similar to that conducted in mice, we used the β‐coefficient for age from a large BMI‐adjusted aging study that employed the same metabolomics platform (Verri Hernandes et al. 2022). In assessing changes in the placebo group from baseline to the first follow‐up at 8 weeks, we observed a non‐significant negative correlation with the metabolomic aging signature, potentially indicating a weak placebo effect (Figure 4A). In contrast, the treatment group receiving 25 mg of losartan daily from baseline to 8 weeks demonstrated a significant negative correlation with the aging metabolomic signature (*ρ* = −0.23, *p* = 5.7e‐3) (Figure 4B). The study design involved dose escalation, with subjects followed up every 8 weeks and the treatment group increasing losartan dosages from 25 mg to 50 mg, and finally 100 mg. We calculated correlations to the “aging metabolomic signature” for each individual at each time point, using their change from baseline as a reference. To assess a potential dose–response relationship, we employed a linear mixed‐effects model, treating subjects as random effects and dose as a second‐order polynomial, which provided the best fit based on Akaike and Bayesian Information Criterion (Figure 4C). The results suggested a “U”‐shaped non‐linear dose–response, with a 50 mg dose of losartan associated with the largest reduction in the metabolomics “aging signature”, while the effect diminished at 100 mg. To identify which specific metabolite classes were most affected by losartan, we constructed similar linear mixed‐effects models to evaluate the impact of losartan dose on each metabolite class. This analysis revealed significant dose–response effects of losartan on phosphatidylcholines and sphingomyelins, with the strongest reductions in serum concentrations observed at a 50 mg dose (Figure 4D,E).

### 3.5 Losartan Treatment Opposes Species‐Divergent Effects of Aging Metabolome

**FIGURE 5:**
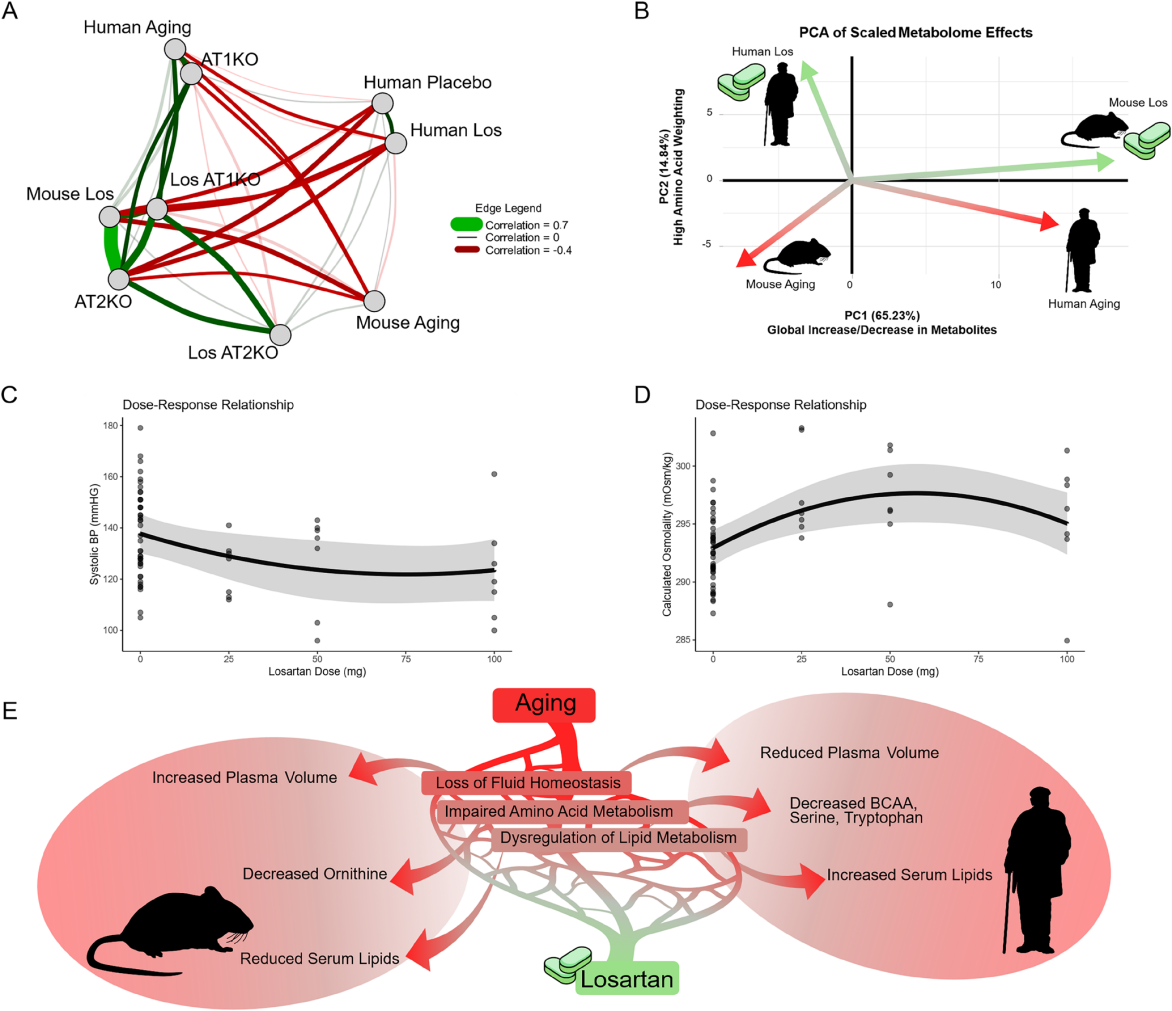
Species‐divergent effects of aging on metabolome reflect shared underlying processes restored by losartan. (A) Correlation network based on effects of labeled conditions as nodes, for all mouse conditions besides aging the comparator group was control aged mice, edges reflect the spearmen's rho between log2FC values of conditions with significant correlations by fdr corrected *p* < 0.05 being opaque and non‐significant being transparent (B) principal component analysis (PCA) of the effects of aging and losartan on mice and humans, values are *z*‐scaled without centering. (C) Dose response of systolic blood pressure modeled with a linear‐mixed effects model with dose treated as second order polynomial and BMI as fixed effects and subject ID as random effects. (D) Dose response of calculated osmolality modeled with a linear‐mixed effects model with dose treated as second order polynomial and BMI as fixed effects and subject ID as random effects. (E) Model of cross‐species serum metabolome rejuvenation effects of losartan‐ aging drives dysfunction in several shared homeostatic and metabolic mechanisms including fluid balance, amino acid, and lipid metabolism, these dysfunctions manifest differently between species, while losartan may help restore these underlying mechanisms resulting in a more youthful metabolic profile in both species.

Finally, we aimed to integrate the results from mice and humans to assess the degree of conservation across species and identify shared or unique mechanisms. We generated a correlation network illustrating the effects of each mouse and human condition on the metabolome (Figure 5A). Notably, we found a significant negative correlation between aging in humans and mice, as well as between losartan treatment effects in both species. Interestingly, losartan treatment in mice closely resembled the effects of AT_2_KO on the metabolome. To further evaluate species‐specific effects of losartan on aging, we performed principal component analysis (PCA) on the effects of aging and losartan treatment in both humans and mice (Figure 5B). The analysis identified a first principal component (PC1) that explained 65.23% of the variance, showing widely distributed loadings across features indicative of a global scaling of metabolites. PC1 decreased with mouse aging (consistent with reductions in most metabolites in aged mouse serum) and increased with human aging (corresponding to increases in most metabolites in aged human serum). PC2, which weighted amino acids more heavily, decreased with age in both species. Despite the differences between human and mouse aging, losartan induced an orthogonal shift in both, driving PC1 up in mice and down in humans, while increasing PC2 in both species. To explore potential mechanisms underlying losartan's effects on the serum metabolome in humans, we evaluated the dose–response effect of losartan on blood pressure and blood osmolality. While we observed a trend toward reduced blood pressure with increasing losartan doses, this effect did not reach statistical significance in our small cohort (Figure 5C). Given losartan's impact on serum metabolite concentrations, we calculated serum osmolality using clinical metabolic panel measures to assess potential shifts in fluid balance. We found a significant effect of losartan dose on serum osmolality, peaking at 50 mg (Figure 5D).

## 4 Discussion

Using targeted metabolomics, we identified significant changes to the serum metabolome of mice with both age and losartan treatment (Figure 1A,B). Our findings regarding age‐related changes to the circulating metabolome of mice correspond to several other published reports using targeted metabolomics, particularly the observation of reduced concentrations of lipid species and the trend toward reduction in most measured metabolites (Eum et al. 2020; Ghorasaini et al. 2021; Zhu et al. 2025). Specifically, we found reductions in SM and LysoPC species, as well as spermidine, which recapitulates findings reported previously in mice (Eum et al. 2020; Zhu et al. 2025). In contrast, we found that losartan treatment resulted in increased serum concentrations of most metabolite measures, and critically this shift was in proportion to that observed with age, resulting in a significant negative correlation (Figure 1B,C). These results suggest losartan treatment can moderately shift the aged metabolome toward a more “youthful” state in mice. Critically, by using mice lacking the AT_1_R or AT_2_R we found that this metabolomic “rejuvenation” effect of losartan required functional AT_2_R or AT_1_R, with absence of either receptor causing loss of losartan's effect, while knock out of either receptor alone, in the absence of losartan, could result in a significant metabolomic “rejuvenation” effect (Figure 1D). These results indicate that losartan's effects on the RAAS are at least partially responsible for its ability to “rejuvenate” the aged mouse metabolome. While aging tended to reduce the concentration of all metabolite classes observed, losartan as well as AT_1_KO and AT_2_KO tended to increase most (Figure 1E). Losartan in particular demonstrated a significant increase in serum amino acid concentration and sphingomyelins not seen in either AT_1_KO or AT_2_KO, providing some evidence of drug‐specific effects.

We identified a similar “rejuvenation” effect of losartan in the cardiac proteome of aged mice (Figure 2). Importantly, this effect was strongest in the whole cell fraction, with increasingly weak effects in plasma membrane and mitochondrial fraction (Figure 2A–C). The weaker effect on the mitochondrial proteome is counterintuitive given that the primary pathway modified by losartan in the whole cell fraction was a reduction in oxidative phosphorylation proteins, a central function of mitochondria. Additionally, of the 11 individual proteins significantly different in whole cell fraction with both age and losartan, 6 were proteins associated with the mitochondrial inner membrane: Cox6b, Coq8a, Phb2, Ndufv2, Coq10b, and Crat (Table S1). Two potential explanations for these observations are: (1) losartan treatment resulted in a shift in cell composition of the cardiac tissue to include less mitochondria‐rich cells, or (2) that losartan treatment resulted in a reduction in mitochondria per cell. To test the first hypothesis, we performed gene set enrichment analysis (GSEA) using gene sets of marker genes for heart tissue cell types and found no indication of change in markers of cell types (Table S2). This would support the second hypothesis that losartan may have reduced mitochondria per cell in cardiac tissue, resulting in a strong signal for an effect on oxidative phosphorylation in whole cell, but a weakened signal when mitochondrial content is equalized by comparing mitochondrial fractions. Interestingly, it has been reported that in mice, aging leads to an increase in mitochondrial number in the heart, which may partially explain the observed general increase in oxidative phosphorylation proteins we observed with age (Figure 2E) (Vue et al. 2023).

While the present study did not include a full lifespan, we did find a statistically significant improvement in survival of geriatric mice treated for a short duration with losartan (Figure 3). These results appear in line with previous reports of lifespan‐extension in rats given losartan or extended lifespan in mice lacking the AT_1_R (Benigni et al. 2009; Basso et al. 2007). It is worth noting that the interventions testing program of the National Institute on Aging, considered the gold standard in rodent longevity studies, has tested two RAAS targeted therapies to date: the angiotensin converting enzyme inhibitors enalapril and captopril (Harrison et al. 2009; Strong et al. 2022; Jiang et al. 2024). In both cases results were relatively equivocal, with effects appearing weak, though significant depending on the statistical tests used, with the selected protocols (Jiang et al. 2024). The results presented in this paper are unique in that they suggest losartan can reduce mortality rapidly even when started in highly advanced age. There are several limitations to this study, including the use of only male mice, relatively small sample sizes, and the termination of the study before complete lifespan curves could be obtained. Ideally, a larger, longer‐term study including both sexes would be conducted to verify these results.

Analysis of targeted metabolomics data from the serum of pre‐frail older men enrolled in a phase 2 randomized placebo‐controlled dose‐escalation study of losartan indicated a similar metabolic “rejuvenation” effect as observed in mice (Figure 4). This effect was found to be dose‐dependent, with the largest impact at 50 mg and loss of effect at 100 mg (Figure 4C). Notably, losartan displayed particularly strong effects on serum lipids, reducing both phosphatidylcholines and sphingomyelins in a dose‐dependent manner (Figure 4D,E). Unfortunately, given the dose escalation design of the study, it is impossible to separate potential effects of drug dose alone from total time on drug. Future studies would therefore need to be designed to assess timing and dose‐specific effects and provide definitive evidence that the effects reported here are dependent on dose alone.

Correlation network analysis provided valuable insights into the effects of losartan and RAAS perturbation on metabolomic aging across species (Figure 5A). Notably, we found significantly negative correlations between the aging shifts in the human and mouse metabolomes, suggesting that the serum metabolomes of these species move in opposite directions with age. This result aligns, however, with some literature indicating inverted relationships in glucose metabolism with age between humans and mice (Palliyaguru et al. 2021). Like aging, the effects of losartan also exhibited negative correlations between species, indicating significantly divergent responses in mice compared to humans. As an example, losartan reduced phosphatidylcholines and sphingomyelins in humans, while these lipid classes significantly increased in mice (Figure 1E). It is important to note that in blood, most lipids—especially phosphatidylcholines and sphingomyelins—are associated with lipoprotein particles, making their levels highly dependent on total lipoprotein content and composition (Zhao et al. 2024). Previous reports suggest that losartan treatment may alter lipid profiles by decreasing LDL and increasing HDL, which could lead to a reduction in total cholesterol (Kyvelou et al. 2006). Thus, the observed lipid reductions may reflect similar effects on systemic lipid metabolism. Additionally, lipoprotein metabolism in mice differs significantly from that in humans, as mice lack cholesteryl ester transfer protein and have lipoprotein profiles that are predominantly HDL with minimal LDL (Steenbergen et al. 2010). This raises the possibility that the divergent species effects of aging and losartan on serum lipids may be a result of the fundamentally different lipoprotein metabolism between these two species. Another noteworthy finding is that AT_2_KO resulted in metabolomic shifts most like those observed with losartan treatment in aged mice. Given that losartan is a selective AT_1_R antagonist, one might have expected it to resemble AT_1_KO more closely. However, research on AT_2_R is less extensive than that on AT_1_R, and its metabolic roles may still need further exploration.

We also employed principal component analysis (PCA) to assess the species‐specific effects of aging and losartan treatment on the serum metabolome (Figure 5B). This analysis revealed a prominent first principal component (PC1) with widely distributed loadings across metabolites, indicating a global change in metabolite levels and particularly lipids. Notably, this is aligned with our findings that most metabolites decrease with age in mice, while many studies report that metabolites tend to increase concentration with age in humans (Verri Hernandes et al. 2022; Tian et al. 2023; Mutz et al. 2024). As concentration is influenced by both mass and volume, the observed global shift may partly result from changes in organismal fluid balance with age, particularly plasma volume. Existing evidence suggests a plasma volume decrease of 10%–20% in older adults (Davy and Seals 1994; Jones et al. 1997). Conversely, studies indicate mice experience an increase in plasma volume with age, which may contribute to observed reductions in metabolite concentrations (Loeffler and Pantel 1990). To explore indicators of fluid regulation, we analyzed the relationship between losartan dose and blood osmolality. Interestingly, we observed a dose–response effect, with the strongest impact at 50 mg (Figure 5D). Contrary to expectations of decreased osmolality to correspond to overall reductions in metabolite concentration from losartan treatment, we found an increase in osmolality, primarily driven by elevated sodium and blood urea nitrogen (BUN) levels. Given the unique fluid dysregulation associated with aging—characterized by reduced plasma volume, diminished urine concentrating ability, and increased arginine vasopressin (AVP) levels—it may be possible that losartan's effects on renal perfusion led to increased sodium and BUN retention, while elevated AVP contributed to a compensatory increase in plasma volume (Cowen et al. 2013). While these differences in lipoprotein metabolism and fluid regulation may partially explain the species‐divergent impacts of aging on the circulating metabolome, several other critical factors such as metabolic rate, body composition, fasting status, and diet may contribute as well.

In summary, we show evidence that both mice and humans exhibit alterations to serum metabolome with age that are partly “rejuvenated” by losartan treatment. Importantly, these age‐related changes in mice and humans are highly distinct, with mice tending to show declines in most metabolites including lipids and some amino acids like ornithine, while humans tend to show increased concentrations of most metabolites including lipids, though showing some reductions in amino acids like branched chain amino acids, serine, and tryptophan. Our proposed model suggests that these species‐divergent aging effects may be explained by loss in homeostasis of underlying regulatory mechanisms in particular fluid homeostasis, amino acid metabolism, and systemic lipid metabolism (Figure 5E). Losartan may act on these underlying regulatory mechanisms to restore homeostatic regulation, thus normalizing age‐related metabolome changes in both species.

## Author Contributions

R.W., A.L., L.F., R.M., J.D.W., J.L.L., and P.M.A. participated in study concept and design. P.M.A. and J.D.W. acted as the PIs for the studies, and J.L.L. and P.M.A. recruited the participants, monitored participant safety, and obtained the functional, demographic, and serum data. J.D.W. and R.W. maintained the aging animal colonies for the mice used in this study. R.M., R.W., and M.K. were responsible for the acquisition of the metabolomics data. C.U.‐M. was responsible for the acquisition of the proteomics data. M.R.B., P.M.A., and R.M. participated in the analysis and interpretation of the data. All authors participated in drafting the manuscript and approved the final version.

## Funding

This study was supported by the National Institute on Aging (NIA) Older Americans Independence Center (NIA grant P30 AG021334 to Johns Hopkins University), NIA career development award K24AG088484 (PMA) and NIA Translational Aging Research Training Grant 2T32AG058527‐06A1(MRB), as well as the National Institute of Arthritis and Musculoskeletal and Skin Diseases, 5T32AR048522‐20 (MRB).

## Conflicts of Interest

The authors declare no conflicts of interest.

## Supporting information


**Table S1:** Significantly different proteins with age in whole cell cardiac fraction.
**Table S2:** Cell‐type enrichment based on disco database cell type markers.

## Data Availability

The data that support the findings of this study are available on request from the corresponding author. The data are not publicly available due to privacy or ethical restrictions.
