# Supplementary material for: Multi‐Omics Reveals Mechanisms of Metabolic Rejuvenation in Aged Mice and Pre‐Frail Older Men by Losartan
Source: Aging Cell. 2026 Apr 22;25(5):e70498. doi: 10.1111/acel.70498 (PMC13100804; doi:10.1111/acel.70498)
Supplement: Supplementary file 1 — Table S1: Significantly different proteins with age in whole cell cardiac fraction. Table S2: Cell‐type enrichment based on disco database cell type markers. [file ACEL-25-e70498-s001.docx]

sTable 1. **Significantly Different Proteins with Age in Whole Cell Cardiac Fraction**

sTable 2. **Cell-Type Enrichment Based on DISCO database Cell Type Markers**
